# Risk or Return? The Effect of Face Consciousness Influences on the Career Construction of Chinese Rural Dwellers in Urban Areas

**DOI:** 10.3389/fpsyg.2022.870655

**Published:** 2022-05-11

**Authors:** Mingfeng Tang, Fenglian Li, Paul Miesing, Mei Mei, Peng Xu

**Affiliations:** ^1^School of Business Administration, Southwestern University of Finance and Economics, Chengdu, China; ^2^Management Department, School of Business, University at Albany-SUNY, Albany, NY, United States; ^3^School of Management, Chongqing University of Technology, Chongqing, China

**Keywords:** Chinese rural dwelllers, face consciousness, indecisiveness, career adaptability, entrepreneurial intention, career construction

## Abstract

This research improves our understanding of entrepreneurial intention in the Chinese cultural context. Drawing on career construction theory, we received 408 valid surveys from rural Chinese dwellers and examined the relationships rural Chinese have among “face consciousness,” indecisiveness, career adaptability, and entrepreneurial intention. We found that those who are fearful of losing face are less likely to have entrepreneurial intentions, but one’s desire to gain face has no significant direct impact on entrepreneurial intention. In addition, face consciousness and an indecisive personality interact to influence career adaptability and, in turn, entrepreneurial intention. In sum, this study supports the career construction perspective on understanding the formation of entrepreneurial intention and offers theoretical, practical, and policy implications for entrepreneurial career counseling and training.

## Introduction

In recent years, urbanization and industrialization in the world’s emerging economies have greatly changed the dominant lifestyle, culture, and behavior of their citizens. It has been predicted that, by 2050, over two-thirds of the world’s population will reside in urban areas, a 13.4% increase from 2018 (United Nations, Department of Economic and Social Affairs, Population Division, 2019). These changes have an extremely significant impact on rural populations as agricultural land is reduced and residents in these regions adapt to the new turbulent environment that urbanization brings, with many turning to entrepreneurial opportunities. The degree of industrialization and commercialization of rural areas and the loss of farmer’s land are found to influence positively the likelihood of rural people becoming private entrepreneurs (Chen, 2006). A reform based on “voluntary cooperatives” with the emphasis on new business cooperation among villagers in the early 2000s promoted the birth of many township enterprises created by rural capable people. These rural entrepreneurs contribute to the development of rural community prosperity (Lan et al., 2014).

The most important motivator for Chinese rural people to start a business is physiological, including safety concerns (Qing et al., 2020). The global financial crisis in 2008 heavily affected the performance of Chinese export-oriented firms, including township enterprises. Rural migrant workers were often factory-floor people and suffered a lay-offs problem. The government encouraged jobless migrants to return home to farm or start new businesses (Chan, 2010). The current rural revitalization strategy initiated by the Chinese government involves a process of land transfer from rural individual households to new economic bodies (e.g., family farms, farmer cooperatives, or agribusiness firms) (Wu and Liu, 2020), which, on one side, promotes the creation of new business; on the other side pushes land lost farmers to migrate to urban areas.

Due to the limited work opportunities for rural dwellers (e.g., a mismatch between high-tech job requirements and productivity potential), they may often be underemployed or totally unemployed (Folmer et al., 2010). Many facing limited career opportunities might regard entrepreneurship as a crucial channel through which they can improve their social and economic lives (Hisrich et al., 2007; Obschonka et al., 2018). He et al. (2019) point out that China recognizes the importance of entrepreneurship to sustain economic development and is accordingly encouraging and facilitating entrepreneurial activity. Entrepreneurship can also alleviate poverty in less-developed regions (Bruton et al., 2013; Si et al., 2015). Therefore, rural entrepreneurship has attracted considerable research attention in recent years.

Some scholars focus on the contribution of self-efficacy to the relationship between the five-factor model of personality traits and entrepreneurial intention (Wang et al., 2016), while others center on the relationship between the types of entrepreneurial opportunity (self-identified opportunities vs. social network- or government-identified opportunities) and the entrepreneurial performance of peasant entrepreneurs (Wu et al., 2020) or emphasize how different institutional elements (i.e., regulative, normative, and cognitive components) affect the strategic behaviors of Chinese rural entrepreneurs (Yu et al., 2013) or the entrepreneurship mechanism of land-lost farmers (Bao et al., 2016). These studies explore antecedents of entrepreneurial intention or entrepreneurial performance, yet little existing literature is devoted to studying the entrepreneurial intention of rural dwellers who have lost their farmland and have to move to urban areas due to industrialization and urbanization. Whether rural dwellers are able to survive in cities through entrepreneurship or are employed decisively affects social stability and sustainable economic development. Career construction is a must for these rural dwellers to consider. Previous studies show that social cognitive factors, such as personal attributes (Bandura, 1986), readiness, orientation, and information (Kulcsar et al., 2020), as well as career adaptability (Savickas, 2005), influence career behaviors. Rural dwellers in China have been immersed in their local culture and possess specific culture-based personal attributes such as “*Mianzi*” (face). We ask if the view of face has a positive effect on their career construction. Are they capable of coping with unprecedent changes (e.g., loss of farmland, need to survive in cities, etc.)? These questions remain underexplored. As entrepreneurship is often a more personal decision than being employed, it is of great importance to study what factors influence the entrepreneurial intention of Chinese rural dwellers from a career construction perspective.

In the entrepreneurship literature, “intention” is often seen as a proximal predictor of entrepreneurial activities (Kautonen et al., 2015; Atitsogbe et al., 2019; Neneh, 2019; Yu et al., 2020). This is because the formation of intention precedes the actual behavior (Ajzen, 1991; Douglas et al., 2021). In view of its important role in the entrepreneurial career process, researchers have devoted considerable effort to examine the antecedents of entrepreneurial intention (e.g., Wilson et al., 2007; Al-Jubari et al., 2019; Ramos-Rodríguez et al., 2019; Botha and Taljaard, 2021). In a comprehensive review of previous studies on entrepreneurial intention, Liñán and Fayolle (2015) point out that the literature consists of two distinct strands of research: one from social psychology focuses on analyzing the psychological process leading from attitudes and cognitions to effective action (e.g., Bandura, 1986; Ajzen, 1991; Botha and Taljaard, 2021); the other examines the field of entrepreneurship, entrepreneurial events, for example (Shapero and Sokol, 1982). These studies have made major contributions to understanding entrepreneurial intention and, therefore, career paths chosen. Inspired by the extant literature, we adopted career construction theory to explore what social cognitive factors impact the entrepreneurial intention of rural dwellers in Sichuan province, China.

## Career Construction Theory

Traditional career theories include career stage theory (Super, 1980), social learning theory of career decision-making (Mitchell and Krumboltz, 1990), work adjustment theory (Dawis and Lofquist, 1984), and self-efficacy theory to career development (Betz and Hackett, 1981). However, these existing theories do not emphasize that careers are built to express an individual’s self-concept that reinforces his or her goals in the reality of work roles (Lent and Brown, 2013) nor do they consider that career development should be driven by adaptation to the environment (Savickas, 2005). Career construction theory (CCT) is one of the contemporary career theories developed since the mid-2000s and has been widely used to study career adaptability (Baruch and Sullivan, 2022). As entrepreneurship is widely regarded as a career choice (Liguori et al., 2020; Marshall and Gigliotti, 2020), entrepreneurship scholars have emphasized a careers perspective that suggests the decision to start a new venture could be thought of as one of many options individuals consider in structuring a meaningful life (Burton et al., 2016). This perspective argues that career experiences and competences can shape entrepreneurial activity. Indeed, several studies in the field of career psychology have brought career theory into entrepreneurship research to examine the antecedents of entrepreneurial career intention (e.g., Pfeifer et al., 2016; Liguori et al., 2018; Pérez-López et al., 2019) and found that career beliefs and abilities affect the formation of entrepreneurial intention. For instance, Chu et al. (2011) found that entrepreneurs in China’s major industrial cities of Beijing, Shanghai, and Guangzhou were mostly motivated by increasing income, becoming their own boss, and to prove that they can succeed. But because a major problem they encountered was a lack of management training, they suggested that policy makers improve management skills and provide technical assistance.

Despite this entrepreneurial career perspective and the important insights existing research has offered, gaps remain in our knowledge of the entrepreneurial career process. This study seeks to complement earlier work on entrepreneurial intention (e.g., Tolentino et al., 2014; Obschonka et al., 2018) that was rooted in a career construction theory and emphasized the adaptation process. Career construction theory (CCT) emphasizes that individuals construct themselves and their careers with career development driven by adaptation to the environment (Savickas, 2005). Career adaptability is the core psychological construct of CCT, which means “an individual’s resources for coping with current and anticipated tasks, transitions, traumas in his or her occupational roles that, to some degree, large or small, alter his or her social integration” (Savickas and Porfeli, 2012, p. 662). Given that social face consciousness is a critical individual internal cognitive state that influences Chinese people’s intentions and behaviors (Huang et al., 2011; Yu et al., 2019), we aim to explain Confucian culture’s association with entrepreneurial intention. Although scholars have pointed out a relationship before (e.g., Begley and Tan, 2001), this argument has not been fully verified. Moreover, no research to our knowledge has investigated the underlying mechanism through which face consciousness affects entrepreneurial intention.

Compared with urban citizens, rural dwellers tend to be less educated, lack formal working experience, and often have lower income, which make them less self-confident and increase the importance of “Mian Zi.” After losing their farmland, they must consider how to adapt to major changes and consider a future career when moving to the city. Career construction requires career adaptability, meaning being ready to cope with uncertainties from unpredictable shifts in their job and working conditions (Savickas, 1997). Many studies have argued the antecedents and consequences of career adaptability in predicting positive career-related outcomes (Jiang, 2017; Zhuang et al., 2018; Gao et al., 2019; Ayala et al., 2020; Mei et al., 2021; Wang et al., 2021). As Obschonka et al. (2018) have indicated career adaptability is important in responding to the uncertain environment in one’s occupational development. However, the existing literature primarily targets university students as samples and pays little attention to personal traits with local culture characteristics. Consistent with the core construct of CCT-career adaptability (Baruch and Sullivan, 2022), our study’s roots are in the Chinese cultural context and argues that face consciousness (enhancement vs. saving) of rural people will impact career adaptability, in turn, shaping the intent to pursue an entrepreneurship career.

Another purpose of the present study is to see how individual indecisiveness affects the linkages between face consciousness and career adaptability. Indecisiveness has received much attention in the field of career decision-making because it plays a critical role in influencing a person’s decision-making process, with indecisive individuals generally encountering greater difficulty across domains and situations (Ng and Hynie, 2014). As suggested by Burton et al. (2016), entrepreneurship is one of the many complex life-course-related choices. Thus, it is possible that it will be difficult for indecisive individuals to adapt to changing conditions and make decisions on their entrepreneurial careers. If so, it is important to examine how indecisiveness interacts with culturally related personality in choosing an entrepreneurship career. In sum, we integrated career construct theory with the literature on face consciousness to develop an additional perspective on why some rural Chinese dwellers are inclined to be entrepreneurs.

**FIGURE 1:**
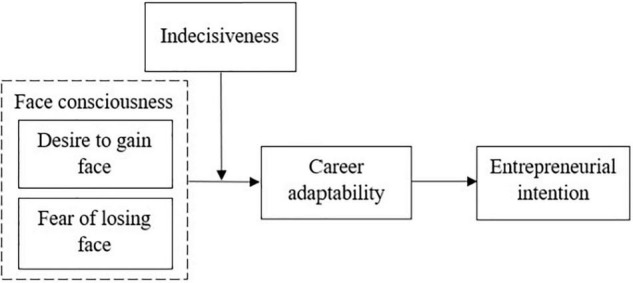
Theoretical model.

Overall, our study makes three contributions to the entrepreneurship literature. First, we conducted an empirical test of whether different dimensions of social face consciousness exert different effects on the career construction process of entrepreneurial intentions. In doing so, we provided a possible explanation of how entrepreneurial intentions are shaped by specific cultural factors. Second, our work expands recent efforts to synthesize the career construction theory, career adaptability, and pieces of entrepreneurial intention literature by identifying the mediating role of career adaptability in the relationship between face consciousness and entrepreneurial intentions. Beyond illustrating the potential effect of individual differences, examining this mediating effect may further provide an in-depth understanding of the career construction processes underlying the emergence of entrepreneurial intentions. Third, we explored differences between “fear of losing face” (risk) and “desire to gain face” (reward) on career adaptability. In this respect, our study contributes to a rather comprehensive understanding of why some rural Chinese dwellers are more susceptible than others to the effects of “face” on career adaptability. The theoretical model is shown in Figure 1.

## Background and Hypotheses

### Face Consciousness and Entrepreneurial Intentions

Culture refers to a set of common values, beliefs, and expected behaviors that influence the dominant personality characteristics of a population (Hofstede, 1980), including how decisions are made and communicated, emotions are expressed, implicit interpersonal relationships and rules, and views about what is right, important, or desirable. These are socially (not genetically) transmitted and homogeneous within societies but heterogeneous between them. Nonetheless, culture is neither unique since other societies can be similar nor is it unimodal since not everyone in a society has the same characteristics and values. Finally, culture can be strong or weak, and it can be dominant or have numerous sub-cultures. Hofstede and Bond (1988) subsequently added traditional Confucian values in explaining entrepreneurial success and economic growth. These factors (similar to the Western Protestant Ethic) include delaying self-gratification for the sake of future benefits; self-discipline, hard work, persistence, and thriftiness; having a sense of shame; supporting interrelatedness through sensitivity to social contacts; and considering education to be very important.

“Face” is a dominant Confucian culture (Zhang et al., 2011) that refers to the positive social value people effectively claim for themselves (Goffman, 1967), reflecting one’s need for recognition and group inclusion (Hwang et al., 2003) that determines the self-image one maintains in the pursuit of acceptance (Huang et al., 2011). Face can be gained, saved, and lost during these social interactions (Wan et al., 2016). While gaining face is not necessary, losing face is a serious matter (Ho, 1976). Face is lost when an individual fails to meet essential requirements expected by virtue of the occupied social position. By connecting people in a specific social structure, face directs their behavior to conform to the local culture as they strive to enhance this positive social value and protect it from being lost (Bao et al., 2003). This belief is like self-efficacy in that it helps to determine one’s choice of activities and environments.

China offers a unique context to study entrepreneurship (He et al., 2019). “*Mianzi*” represents the prestige, honor, and respect brought about by success, higher social status, and ostentatious behaviors before others (Hu, 1944; Zhang et al., 2011). For Chinese rural dwellers, “*Mianzi*” means reciprocity, social appraisal, good reputation, and high social status. Social appraisal arises from personal capability, interactions with local villagers, and performance in rural community affairs (Dong and Guo, 2017). For historical and institutional reasons, rural dwellers do not have industrial jobs and access to (at least basic) social welfare and full citizenship that urban people do (Chan, 2010). They live in rural communities, which are comparatively less open compared with urban areas but preserve a more traditional Confucian culture. This is partially related to their affiliation to farmland for generations. However, the large-scale migration of rural dwellers to urban areas since the 1980s freed them from their attachment to farmland and created a desire to fend for themselves. Since more money or wealth is widely viewed as having more “*Mianzi*” (Dong and Guo, 2017), entrepreneurs gain “*Mianzi*” (e.g., reputation, social status, high social appraisal, honor, prestige, respect) as they return home, and their strong personal capability to earn more money contributes to rural community prosperity.

East Asian culture accepts the paradox of “yin” and “yang” where opposites comfortably co-exist. Similarly, there is a duality to “face consciousness”: the simultaneous desire to gain face and fear of losing face (Zhang et al., 2011). In the present study, we propose that each dimension of face consciousness is simultaneously present and has a different influence on entrepreneurial intentions. On the one hand, the *desire to gain face* (reward) will drive some to be an entrepreneur. China’s recent economic policies intending to facilitate mass entrepreneurship and innovation should motivate individuals to choose a socially encouraged entrepreneurial career path that will secure social legitimacy and gain face. For instance, Lau and Busenitz (2001) found that Chinese entrepreneurs’ need for achievement determined their growth intentions. Additionally, entrepreneurship can be a feasible way to raise one’s earnings potential (Murnieks et al., 2020). Compared with “working for others,” “being a boss” means certain power, which is a career choice that can improve social status for those who otherwise have limited employment opportunities. In this respect, the desire to gain face may create outcome expectations (e.g., higher income, self-esteem, and self-actualization) that lead to career goals (e.g., trigger entrepreneurial intention) in China.

On the other hand, the *fear of losing face* (risk) will hinder the intention to choose entrepreneurship as a career. Empirical studies suggest that individuals who are afraid of being embarrassed or do not want to lose face tend to avoid risky events. For instance, a study in the Chinese cultural context found that individuals tend to decrease help-seeking behaviors to avoid being rejected by others (Lei and Tang, 2015). Due to the high uncertainty of entrepreneurship success (Wennberg et al., 2013), those choosing an entrepreneurial career are vulnerable to business failure. The more people are afraid of failure, the less they are willing to start a business (Kong et al., 2020). In a shame-based society like China, failure is a serious threat to saving face (Begley and Tan, 2001). In this regard, a person who has a deep fear of losing face would anticipate such undesirable results as lost earnings, ridicule, and family conflicts. These potential outcomes would make them hesitant or even fearful of starting a new business. Taken together, we propose the following hypotheses:

*Hypothesis 1a: Desire to gain face is positively associated with entrepreneurial intentions.*

*Hypothesis 1b: Fear of losing face is negatively associated with entrepreneurial intentions.*

### Face Consciousness and Career Adaptability

Individual differences regarding a flexible personality or willingness to change denote those who are more likely to adapt (Savickas and Porfeli, 2012; Guan et al., 2017). Face consciousness is considered as a relatively stable personality that influences Chinese cognitive and affective reactions when they interact with others (Chou, 1996; Cheung et al., 2008). Career adaptability is defined as “self-regulation strengths or capacities that a person may draw upon to solve the unfamiliar, complex, and ill-defined problems presented by developmental vocational tasks, occupational transitions, and work traumas” (Savickas and Porfeli, 2012, p. 663). It is measured by concern (such as for one’s future), control (making one’s own decisions), curiosity (exploring opportunities while developing possible selves), and confidence (overcoming obstacles) (Savickas and Porfeli, 2012). In view of the different behavioral implications of “desire to gain face” and “fear of losing face,” we argue that these two distinct face needs have different effects on career adaptability. Individuals with a need for face would be motivated to consider their future, expect positive outcomes, make their own decisions, and take actions that will manage and disseminate positive images, display their strengths, and behave in line with social norms so as to attain social acclaim (Huang et al., 2011). Hence, those concerned with gaining face have a high need for achievement, are willing to take risks to achieve their goals, and may appear assertive and confident (Chou, 1996). For them, a dynamic environment is not always a threat but offers good business opportunities. Therefore, we can conclude that those who are having a high face-gaining orientation tend to actively engage the changing environment, set challenging goals, engage in self-promoting behaviors to be recognized by others, and solve occupational transition problems. In sum, the “desire to gain face” is a motivational factor that facilitates adaptability.

Yet not everyone is eager to gain face. When Chinese people cannot gain face during social interactions, at least they protect its damage (Ho, 1976) since losing face may induce such negative emotions as shame and depression (Begley and Tan, 2001; Huang et al., 2011). Because individuals who are afraid to lose face are sensitive to the potential threat of being looked down upon by others, they tend to exhibit cautious and withdrawal behaviors (Chou, 1996). Those who fear losing face will avoid sharing information and knowledge, thereby hindering career adaptability (Gong and Li, 2019). For example, empirical studies have shown conscious face saving to be negatively related to knowledge sharing intentions (Huang et al., 2011). Students have been reluctant to seek feedback in class because of their fear of losing face (Hwang et al., 2003). As the 21st century experiences more rapid changes than ever, uncertainty is undoubtedly higher than before. The advancement of information technology accelerates the transfer of negative stories further and faster than before. Fear of losing face causes people to be concerned about their future, lack courage and confidence to explore business opportunities, incapable of making their own decisions, unable to take risks, and hinder exchanges of information and knowledge; all of which make people unwilling to adapt to the changing environment. Therefore, we propose that:

*Hypothesis 2a: Desire to gain face is positively associated with career adaptability.*

*Hypothesis 2b: Fear of losing face is negatively associated with career adaptability.*

### Mediating Role of Career Adaptability

During the urbanization process, rural dwellers encounter a series of challenges such as losing their farming lands, integrating into urban life, and career transitions. These changes require them to adapt to their surroundings. Given that China has increasingly emphasized rural entrepreneurship (Yu et al., 2013) as an important and effective way to reduce poverty and increase well-being (Markussen et al., 2017; Lin et al., 2020), this study focuses on entrepreneurship as an indicator of rural dwellers adapting to a vital career pathway and benefiting from China’s economic growth.

Career adaptability refers to psychosocial resources that enable people to cope with current and anticipated challenges, transitions, or trauma in changing contexts (Savickas, 1997, 2005, 2013; Savickas et al., 2009). It is vital for individuals to develop and determine the optimal strategy to guide their adaptive behaviors (Rudolph et al., 2017; Johnston, 2018; Peng et al., 2021). Indeed, recent studies have argued that career adaptability might be a significant predictor of entrepreneurial intentions because highly adaptable individuals possess self-regulation strengths that enable them to better identify business opportunities, mobilize resources, and fit into uncertain circumstances (Tolentino et al., 2014). In their research from student samples, Tolentino et al. (2014) demonstrated that entrepreneurship is a vocational strategy that can be driven by individuals’ career adaptabilities. Moreover, the four types of adaptability resources (i.e., concern, control, curiosity, and confidence) reflect self-efficacy and a tendency to make the effort to prepare for and explore a satisfactory career path (Savickas and Porfeli, 2012). In this respect, it is plausible that those who are better able to adapt will more proactively explore and prepare for entrepreneurship than those with lower career adaptability. We believe that career adaptability will facilitate entrepreneurial intention and, therefore, hypothesize the following:

*Hypothesis 3: Career adaptability is positively associated with entrepreneurial intentions.*

Career adaptation involves a sequential process through which adaptive personality traits impact career adaptability resources, in turn promoting responses and beliefs, enabling individuals to cope with changing environments (Hirschi et al., 2015; Pajic et al., 2018). Combined with our arguments above for the link between face consciousness and career adaptability (Hypothesis 1) and the link between career adaptability and entrepreneurial intention (Hypothesis 2), we further propose the following hypothesis concerning the mediating role of career adaptability:

*Hypothesis 4: Career adaptability mediates the relationship between face consciousness and entrepreneurial intentions.*

### Moderating Role of Indecisiveness

Indecisiveness is a trait that refers to the pervasive and chronic difficulties in making decisions, regardless of its significance, when individuals are in a mix of good and bad situations (Crites, 1969). Indecisiveness is correlated with certain personality characteristics, such as low self-confidence, low self-esteem, helplessness, and a sense of contradiction and frustration (Salomone, 1982; Germeijs and De Boeck, 2002; Öztemel, 2014; Lo Cascio et al., 2016). This premise implies that indecisive individuals often feel anxious and fearful when facing opportunities and choices, so they are typically labeled as undecided in making all sorts of life decisions (Gati et al., 2011), and indecisiveness may impede effective actions (Appel et al., 2021).

In the present study, we argue that indecisiveness will interact with face consciousness to impact the development of career adaptability. Specifically, indecisiveness could harm one’s adaptation results, thereby diminishing the quality of life (Taillefer et al., 2016). When individuals have a strong desire to gain face, the hesitancy caused by indecisiveness will deepen one’s feeling of contradiction, in turn frustrating and lowering their self-esteem, which is not conducive to career adaptability (Cai et al., 2015). In contrast, a decisive person is more likely to be proactive and confident in making career choices (Germeijs and De Boeck, 2002) so that those with a face-gaining orientation might take active actions and make high-quality decisions (Gati et al., 2011; Kokkoris et al., 2019). We thus propose the following:

*Hypothesis 5a: Indecisiveness moderates the relationship between a desire to gain face and career adaptability, being more positive when it is low than when it is high.*

Indecisiveness can also impact the relationship between fear of losing face and career adaptability. Specifically, indecisive individuals are considered to be vulnerable to adaptive psychological problems like anxiety (Germeijs and Verschueren, 2011), neuroticism (Jackson et al., 1999) and non-clinical depression (Di Schiena et al., 2013). These individuals are more likely to interpret uncertainty as a threat, thus delaying or even avoiding making decisions in complex and ambiguous situations (Rassin and Muris, 2005). Such fear of uncertainty will be magnified when a person is simultaneously afraid of losing face and is indecisive, resulting in unwillingness to change the *status quo* and hesitance in making decisions (Sautua, 2017). Such a person is less likely to show initiative, hindering career adaptability. Therefore, we propose:

*Hypothesis 5b: Indecisiveness moderates the relationship between fear of losing face and career adaptability, being more negative when it is high than when it is low.*

## Research Method

### Sample and Data Collection

Elston and Weidinger (2019) argue for analyzing rural regions in large diverse markets such as China. In fact, they conclude that the relatively higher levels of necessity-based entrepreneurship in the countryside may explain some of the regional differences in entrepreneurial activity. To test our hypotheses, we recruited the participants in Sichuan Province in southwest China. With 83.75 million regular inhabitants, Sichuan is China’s fourth most populous province. Although from a specific context, Sichuan is a representative province for conducting research about the career development process rural dwellers face since this sample reflects rural entrepreneurship. Historically known as China’s breadbasket, it is one of the leading Chinese provinces in terms of agricultural production and, hence, dominated by farm workers. In recent years, rapid urbanization and industrialization have brought tremendous changes to its rural areas. Until 2017, the urbanization rate was 50.79%, passing 50% for the first time (Hou and Fan, 2019).

Our research sample targets rural dwellers in Sichuan who have lost their farmland and have recently moved to urban areas and have not created their business yet. This study instrument was approved by the academic ethics committee of the Southwestern University of Finance and Economics. Thanks to the help of the local government demolition bureau, we obtained living addresses of rural dwellers, randomly selected one of the living communities (Yucheng District in Ya’an, which is located in the west of Sichuan Basin and reachable by public transportation), and then made a one-on-one household survey, with each family selecting either one male host, one female host, or both to participate in the early stages of the investigation on January 06, 2020. However, the door-to-door survey was interrupted due to the outbreak of COVID-19. Because only 56 valid surveys out of 100 were returned, we shifted on January 20, 2020 from the on-site survey to an online survey through a widely used platform in China called “Wen Juan Zhi Xing.” We employed a snowball sampling technique through referrals among the people who had completed the questionnaire in January. The criteria for the participants are they must be Chinese rural dwellers; understand Mandarin; have access to the Internet; and are not entrepreneurs at the survey moment. Those who did not meet these criteria were excluded from participation. For assuring the participants were able to answer the digital survey, we left a contact cellphone number in the survey so they could inquire if needed. The referrals familiar with the online survey also agreed to help these participants answer an E-VERSION survey. The participation was voluntary, with all the participants being informed about the purpose of the study and that the data would remain anonymous. To motivate participation, those who were completing the questionnaire received a modest incentive (i.e., a small gift or minor cash payments) after submitting the survey. As of August 31, 2020, 408 valid questionnaires in total were included in the final analysis (a response rate of 92.1%), excluding those with missing information, same answers, or completed in too short a time. We believe the high rate of response is due to the easy-to-understand survey content, our participation incentive, and the high voluntary participation of rural dwellers.

### Measures

#### Dependent Variable

A six-item scale developed by Liñán and Chen (2009) was used to measure entrepreneurial intentions. This scale is closely related to entrepreneurial behavior, such as “I am ready to do anything to be an entrepreneur; I will make every effort to start and run my own firm”; and it has been well validated in the Chinese context (Lin and Si, 2014). Example items included “I very seriously thought of starting a firm,” “My professional goal is to become an entrepreneur,” and “I am determined to create a firm in the future.” The respondents were asked to rate their entrepreneurial intentions on a five-point Likert-type scale, ranging from 1 = *strongly disagree* to 5 = *strongly agree*. The Cronbach’s alpha for this scale was 0.909.

#### Independent Variables

We used the 11-item scale developed by Zhang et al. (2011) to measure social face consciousness. It has been adopted to measure face consciousness of Chinese people (e.g., Du and Xu, 2014; Lei and Tang, 2015). This scale consists of two distinct dimensions that are understood to capture an individual’s orientation/tendency to manage his or her social “face.” Of the 11 items, six are used to assess the desire to gain face (e.g., “I hope people think that I can do better than most others,” “It is important for me to get praise and admiration”) and five are used to assess the fear of losing face (e.g., “I try to avoid letting others think that I am ignorant, even if I really am,” “I do my best to hide my weakness before others”). All items were on a five-point Likert-type scale with anchors from 1 = *strongly disagree* to 5 = *strongly agree*. The Cronbach’s alphas for desire to gain face and fear of losing face were 0.813 and 0.764, respectively.

#### Moderator

We used a four-item subscale from the career decision difficulties questionnaire (CDDQ) (Gati et al., 1996) to measure indecisiveness. Example items included “A general difficulty in making decisions” and “A general need for confirmation and support for decisions.” The respondents were asked to rate the extent to which each statement describes them on a five-point Likert-type scale, ranging from 1 = *strongly disagree* to 5 = *strongly agree*. The Cronbach’s alpha was 0.724.

#### Mediator

To measure career adaptability, we used the career adapt-abilities scale-short form (CAAS-SF) developed from Savickas and Porfeli’s (2012) widely used CAAS scale (Maggiori et al., 2017) that has been adopted for the Chinese context (Cai et al., 2015; Jiang, 2017). This brief version scale has 12 items that are divided into four dimensions: concern, control, curiosity, and confidence. Example items included “Preparing for the future” (concern), “Making decisions by myself” (control), “Observing different ways of doing things” (curiosity), and “Learning new skills” (confidence). Responses were given on a five-point Likert-type scale (from 1 = *not strong* to 5 = *strongest*). The Cronbach’s alpha for overall career adaptability was 0.808. Before formally administering the survey for our study, we had launched a pre-survey of 50 randomly selected rural dwellers and checked whether they understood the scale items. Based on their feedback, we made minor modifications of some words, which made the scales easier to be understood without deviating from the original meaning.

#### Control Variables

We controlled for a series of relevant variables that may explain some differences in entrepreneurial intention. First, previous research on entrepreneurial intention suggests that it might be associated with an individual’s demographic characteristics (Wilson et al., 2007). Gender was dummy coded with women as “0” and men as “1.” Age was coded as “1” for those 18–25 years old, “2” for those 26–30 years old, “3” for those 31–35 years old, “4” for those 36–40 years old, “5” for those 41–45 years old, and “6” for those over 45 years old. Marital status was dummy coded with single being a “0” and married being a “1.” Education was coded as “1” for those who finished junior middle school or below, “2” for those who finished high school, “3” for those who had a junior college degree, and “4” for those who had a bachelor degree or higher.

Second, we controlled for entrepreneurial education, prior entrepreneurial experience, and family business exposure because they are suggested to be important in forming entrepreneurial intentions (Stuart and Abetti, 1990; Bonesso et al., 2018; Bloemen-Bekx et al., 2019). Entrepreneurial education participation was measured using the following question: “Have you ever attended any entrepreneurship courses or training?” (no = 0, yes = 1). Following prior research (Hmieleski and Baron, 2009), individuals’ previous entrepreneurial experience was measured by a single item, asking the respondents to report the number of new businesses they started. Family business exposure was measured using the following question: “Have any of your family members ever started a new business?” (no = 0, yes = 1).

### Common Method Bias

Because we collected data from a single source and used self-reported questionnaires, we employed Harman’s one-factor test to ensure that common method bias (CMB) did not pose a serious threat to this study. Loading all items into a principal component factor analysis, the first emerging unrotated factor accounted for 29% of the total variance, much lower than the 50% threshold. This indicates no major sign of common method bias in this study.

## Results

### Descriptive Statistics and Confirmatory Factor Analysis

We used SPSS 25.0 for the descriptive statistical analysis among variables. Of the 408 valid questionnaires, 41.9% were farm workers and 58.1% were unemployed. Means, standard deviations, and Pearson correlations between study variables are reported in Table 1. Nearly half were male, and most participants were married. In terms of age, the largest sub-group had one-third in their late twenties. Regarding education, one-third had at least some college education and 17.4% indicated that they had participated in entrepreneurship training programs and courses. In addition, over half of the participants had family members who started a new business, and over half had previous entrepreneurial experience.

**TABLE 1:** Means, standard deviations, and Pearson correlations among variables.

Variable	*M*	*SD*	1	2	3	4	5	6	7	8	9	10	11
**Control**													
(1) Gender	0.480	0.500											
(2) Age	3.040	1.674	0.044										
(3) Marital status	0.620	0.499	0.048	0.115**									
(4) Education	2.200	1.140	−0.209**	−0.491**	−0.179**								
(5) EE	0.170	0.380	0.003	0.017	−0.017	−0.006							
(6) PE	1.150	1.353	0.017	0.109*	−0.266**	−0.020	0.139**						
(7) FB	0.530	0.499	−0.122*	0.088	0.066	−0.003	0.086	0.065					
Independent													
(8) DGF	3.124	0.751	−0.022	−0.082	0.035	0.063	−0.020	0.007	0.103*				
(9) FLF	2.808	0.738	0.044	−0.102*	0.006	0.049	0.035	−0.107*	0.031	0.403**			
Moderator													
(10) IND	3.061	0.845	−0.010	−0.042	−0.028	−0.080	0.041	0.007	−0.064	−0.058	0.201**		
Mediator													
(11) CA	3.778	0.617	0.011	−0.012	−0.059	0.211**	0.102*	0.107*	0.072	0.219**	−0.189**	−0.136**	
Dependent													
(12) EI	3.072	0.819	−0.056	−0.047	−0.078	0.037	0.218**	0.275**	0.134**	0.017	−0.183**	−0.063	0.334**

*N = 408. Gender (women = “0” and men = “1”); age (“1” = 18–25 years old, “2” = 26–30 years old, “3” = 31–35 years old, “4” = 36–40 years old, “5” = 41–45 years old, “6” = over 45 years old); marital status (single = “0” and married = “1”); education (“1” = junior middle school or below, “2” = high school, “3” = junior college, and “4” = bachelor’s degrees or higher); EE, entrepreneurship education (“Have you ever attended any entrepreneurship courses or training?” yes = 1); PE, prior entrepreneurial experience (number of new businesses started); FB, family business exposure (“Have any of your family members ever started a new business?” yes = 1); DGF, desire to gain face (from 1 = strongly disagree to 5 = strongly agree); FLF, fear of losing face (from 1 = strongly disagree to 5 = strongly agree); IND, indecisiveness (from 1 = strongly disagree to 5 = strongly agree); CA, career adaptability (from 1 = not strong to 5 = strongest); EI, entrepreneurial intentions (from 1 = strongly disagree to 5 = strongly agree).*

*p < 0.05, **p < 0.01.

The correlations among the five focal variables were basically in the expected direction. The two face-conscious independent variables are significantly correlated. The desire to gain face is positively correlated with career adaptability, whereas fear of losing face is negatively correlated with career adaptability and entrepreneurial intention. Moreover, fear of losing face is highly correlated with indecisiveness. Career adaptability is positively correlated with entrepreneurial intention, but the relation between desire to gain face and entrepreneurial intention is not significant. Among the control variables, only entrepreneurial education participation, prior entrepreneurial experience, and family business exposure are all positively correlated with entrepreneurial intention. Entrepreneurial education participation and prior entrepreneurial experience are also highly correlated with each other but not with family business exposure. Career adaptability is positively correlated with increasing education and decisiveness. Finally, career adaptability and entrepreneurial intensions are highly correlated.

Before testing the hypotheses, we used AMOS21.0 to conduct confirmatory factor analysis (CFA) to evaluate the discriminant validity of the core (non-control) variables. The CFA results indicate that the hypothesized five-factor model in Figure 1 (desire to gain face, fear of losing face, indecisiveness, career adaptability, and entrepreneurial intention) shows an acceptable fit to the data. We compared the hypothesized five-factor model with alternative models, including a single-factor model that grouped all variables into one factor; a two-factor model in which face consciousness and indecisiveness were grouped as one factor, and career adaptability and entrepreneurial intention were grouped as another factor; a three-factor model in which face consciousness and indecisiveness were grouped as one factor; and a four-factor model in which the two dimensions of face consciousness were grouped as one factor. The results in Table 2 show that the five-factor model is better than the other four models.

**TABLE 2:** Confirmatory factor analysis results for the measurement models.

Model	χ*^2^*	*df*	χ*^2^/df*	CFI	TLI	IFI	RMSEA
Hypothesized five-factor model	402.466	109	3.692	0.913	0.891	0.914	0.081
Four-factor model: (DGF + FLF), IND, CA, EI	598.705	113	5.298	0.856	0.827	0.857	0.103
Three-factor model: (DGF + FLF + IND), CA, EI	855.363	116	7.347	0.781	0.743	0.782	0.125
Two-factor model: (DGF + FLF + IND), (CA + EI)	1380.894	118	11.702	0.625	0.568	0.627	0.162
One-factor model: (DGF + FLF + IND + CA + EI)	1756.129	119	14.757	0.514	0.445	0.517	0.184

*N = 408. DGF, desire to gain face; FLF, fear of losing face; IND, indecisiveness; CA, career adaptability; EI, entrepreneurial intentions.*

### Test of Direct and Mediator Effects

We examined the direct effect of face consciousness on entrepreneurial intention. (Hypotheses 1a and 1b). The results of Model 6 in Table 3 show that the effect of desire to gain face is not significant in explaining entrepreneurial intentions (β = 0.003, *ns.*). Hence, H1a is not supported. In stark contrast, the results of Model 8 reveal a significantly negative effect that fear of losing face has on entrepreneurial intention (β = −0.182, *p* < 0.001), supporting H1b.

**TABLE 3:** Results for the hierarchical regression analysis of direct, mediator, and moderator effects.

Variable	Career adaptability				Entrepreneurial intention				
	Model 1	Model 2	Model 3	Model 4	Model 5	Model 6	Model 7	Model 8	Model 9
**Control variables**									
Gender	0.071	0.082	0.076	0.075	−0.068	−0.049	−0.073	−0.039	−0.063
Age	0.125*	0.090	0.107*	0.078	−0.123*	−0.089	−0.127*	−0.105	−0.128*
Marital status	−0.016	−0.008	−0.021	−0.010	−0.003	−0.009	−0.011	−0.015	−0.019
Education	0.273***	0.282***	0.268***	0.271***	−0.097	−0.012	−0.102	−0.010	−0.091
Entrepreneurship education	0.091	0.094	0.083	0.096*	0.149**	0.176***	0.147**	0.185***	0.159***
Prior entrepreneurial experience	0.077	0.060	0.098*	0.076	0.228***	0.253***	0.227***	0.232***	0.214***
Family business exposure	0.037	0.070	0.020	0.061	0.086	0.107*	0.950*	0.118*	0.098*
Independent variables									
Desire to gain face	0.212***		0.175***			0.003	−0.062		
Fear of losing face		−0.197***		−0.177***				−0.182***	−0.126**
Mediator									
Career adaptability					0.308***		0.325***		0.267***
Moderator									
Indecisiveness			−0.095*	−0.086					
Interaction effects									
Desire to gain face × Indecisiveness			−0.166**						
Fear of losing face × Indecisiveness				−0.107*					
*R* ^2^	0.124	0.118	0.160	0.134	0.216	0.130	0.223	0.162	0.235
Adj. *R*^2^	0.107	0.100	0.139	0.113	0.200	0.112	0.205	0.145	0.217
*F*	7.077	6.679	7.549	6.163	13.721	7.359	12.581	9.534	13.421

N = 408. *p < 0.05, **p < 0.01, ***p < 0.001.

To examine the mediation model, we employed hierarchical regression analysis to test the relationship of face consciousness with career adaptability and the relationship of career adaptability with entrepreneurial intention as well as bootstrapping analysis of the significance of indirect effects. The results of Model 1 indicate that the desire to gain face on its own has a positive and significant effect on career adaptability (β = 0.212, *p* < 0.001), supporting H2a. When we regressed entrepreneurial intention on the control variables and career adaptability in Model 5, the results showed that career adaptability had a significant and positive effect on entrepreneurial intention (β = 0.308, *p* < 0.001). Thus, H3 was supported. When we regressed entrepreneurial intention on the control variables, desire to gain face, and career adaptability, the results in Model 7 suggest that career adaptability plays a mediating role between desire to gain face and entrepreneurial intention (β = 0.325, *p* < 0.001). Further examining the mediating effect by the PROCESS procedure (Model 4), the bootstrapping results confirm the indirect effect of career adaptability in linking the desire to gain face with entrepreneurial intention (see Table 4). Overall, H4a is supported.

**TABLE 4:** The bootstrap result on mediating effect by the PROCESS procedure (Model 4).

	Effect	Boot se	95% Confidence Intervals	
			Lower limit	Upper limit
Indirect effect of desire to gain face on entrepreneurial intention through career capability	0.075	0.029	0.028	0.138
Indirect effect of fear of losing face on entrepreneurial intention through career capability	−0.062	0.024	−0.116	−0.022

We next examined the mediating role of career adaptability between fear of losing face and entrepreneurial intention. As shown in Model 2, fear of losing face is negatively related to career adaptability (β = −0.197, *p* < 0.001), supporting H2b. When we regressed entrepreneurial intention on the control variables, fear of losing face, and career adaptability, the results in Model 9 suggest that career adaptability plays a mediating role between fear of losing face and entrepreneurial intention (β = 0.267, *p* < 0.001). Further examining the mediating effect by the PROCESS procedure (Model 4), the bootstrapping results confirm the indirect effect of career adaptability in linking the desire to gain face with entrepreneurial intention (see Table 4). Hence, H4b was supported.

### Test of Moderator Effects

**FIGURE 2:**
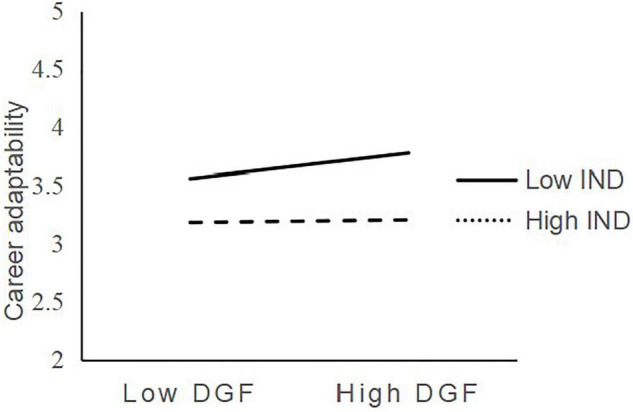
Interaction of desire to gain face and indecisiveness predicting career adaptability.

Hypotheses 5a and 5b predicted the moderating role of indecisiveness on the relationship between face consciousness and career adaptability. We followed the widely used three-step procedure (e.g., Jiang, 2017) to test for these effects. All focal predictors were mean centered before the analysis (Aiken and West, 1991). The results of Model 3 display a significant and negative interaction of desire to gain face and indecisiveness in predicting career adaptability (β = −0.166, *p* < 0.01), suggesting that indecisiveness lessened the positive effect of desire to gain face on career adaptability. Figure 2 depicts the simple slopes for the interaction effect between desire to gain face and career adaptability, which is significant when decisive (*b* = 0.253, *SE* = 0.045, *p* < 0.001) but not when indecisive (*b* = 0.035, *SE* = 0.055, *ns*). This is consistent with H5a.

**FIGURE 3:**
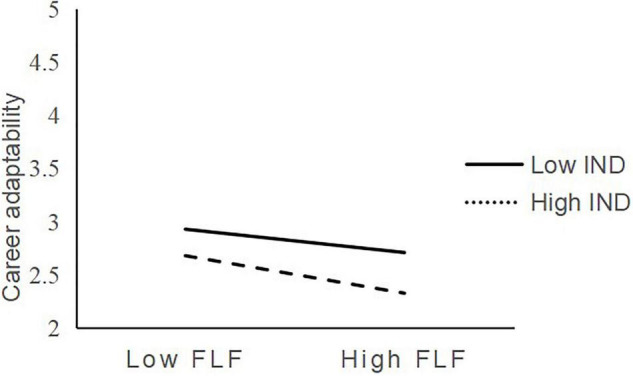
Interaction of fear of losing face and indecisiveness predicting career adaptability.

**FIGURE 4:**
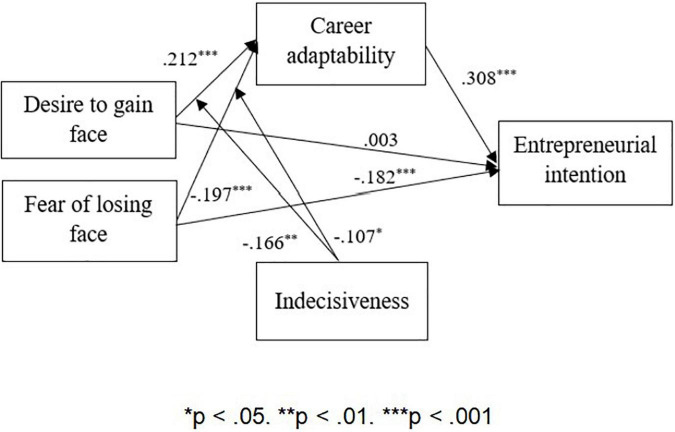
Summary of the regression results.

Likewise, the results in Model 4 show that the interactive effect of fear of losing face and indecisiveness on career adaptability is negative and significant (β = −0.107, *p* < 0.05). As indicated in Figure 3, the relationship between fear of losing face and career adaptability is more negative when indecisiveness is high (*b* = 0.225, *SE* = 0.052, *p* < 0.001) than when indecisiveness is low (*b* = −0.072, *SE* = 0.054, *ns*). Hence, H5b was supported. The regression results are summarized in Figure 4.

The major plausible relationships of these results are as follows: (1) Risk of losing face has a significant negative influence on entrepreneurial intention, but the reward of gaining face by itself, while positive, is not significant; (2) Face consciousness has a significant impact on career adaptability, with risk of losing face being negative and reward of gaining face being positive; (3) This influence of face consciousness on career adaptability is reduced when an individual is indecisive regardless of fear of risk or desire of reward; and (4) Career adaptability has a highly significant influence on entrepreneurial intention.

## Discussion

Career construction theory provides a theoretical framework for studying the individual entrepreneurial career development process. However, there is relatively little empirical work that has adopted this perspective to explain the formation of entrepreneurial intention [notable exceptions being Tolentino et al. (2014) and Obschonka et al. (2018)]. Integrating insights from the career construction literature, we proposed and found that social face consciousness impacts career adaptability, which, in turn, influences entrepreneurial intent. Furthermore, we demonstrated that face consciousness and an indecisive personality interact to influence the development of career adaptability. We discuss the theoretical, practical, and policy implications of these results.

### Theoretical Implications

Our research extends entrepreneurial intention and career construction theory in several important ways. First, by investigating the effect of face consciousness (i.e., desire to gain face and fear of losing face) on entrepreneurial intention, we add to the existing knowledge of the antecedents of entrepreneurial intent. Although prior research has provided valuable insights into its drivers (e.g., Tolentino et al., 2014; Pfeifer et al., 2016; Pérez-López et al., 2019), relatively little attention has been focused on the influence of specific cultural constructs. Culture contributes to shaping an entrepreneurial mindset and influences regional rates of entrepreneurship (Hayton et al., 2002; Schlaegel et al., 2013; Karimi et al., 2021). Our survey results found that distinct face needs will affect entrepreneurial intentions in different ways. Specifically, fear of losing face will significantly hinder entrepreneurial intention, whereas the desire to gain face, while positive, is not significant in explaining entrepreneurial intention. This suggests that at least for Chinese rural dwellers’ entrepreneurial career construction, fear of failure outweighs the possibility of success. This finding is consistent with prior literature, positing that face-gaining consciousness would be less primary and less important than face-saving consciousness in an Asian culture (Chou, 1996; Kim and Nam, 1998) and enriches the existing career construction theory.

We also contribute to the entrepreneurial intention literature by identifying career adaptability as a mediating mechanism that links face consciousness with entrepreneurial intention. Although career adaptability is important in shaping one’s entrepreneurial intention (e.g., Tolentino et al., 2014), empirical studies that directly apply a career construction perspective to examine the relationship between career adaptability and entrepreneurial intention remain scarce. Our work reinforces evidence from prior studies that there is a positive relationship between career adaptability and entrepreneurial intention. Furthermore, our study confirms the mediating role of career adaptability in translating face consciousness into entrepreneurial intention. This is meaningful because this study shows how a career perspective offers additional insights for understanding the entrepreneurial process.

Finally, this study examined indecisiveness as a moderator to explain when face consciousness influences career adaptability and the entrepreneurial process. While prior research has discussed the significantly negative role of indecisiveness in general career decisions (e.g., Öztemel, 2014; Lo Cascio et al., 2016), there has been rare attention paid to how individuals’ indecisiveness can exert a negative effect on entrepreneurial decisions and processes. We extend this line of research and demonstrate that indecisiveness amplifies the negative effect that fear of losing face has on entrepreneurial intention and lessens the positive effect that desire to gain face has on entrepreneurial intention. This finding further attests to the important role of individuals’ adaptive personality in forming entrepreneurial intention.

### Practical and Policy Implications

The results of our study offer several meaningful implications. Serial entrepreneurs in the West are willing to take risks to achieve success, hoping that the one big hit compensates for the many small misses. Moreover, bankruptcy laws minimize the stigma of failure. Either way, they accept risk and manage to mitigate losses in pursuing abnormal returns. On the other hand, we show that China’s rural dwellers are more concerned about “losing face,” especially when indecisive in forming entrepreneurial intentions. Therefore, entrepreneurship education courses should be connected to a specific cultural context for those Chinese regions seeking to stimulate the growth of new businesses. For example, role-model exposure that allows prospective entrepreneurs to learn from other failure experiences can mitigate the fear and embarrassment of losing face. Showing the universality and even benefits of entrepreneurial failure (Lee and Miesing, 2017) would signal that, while risk trumps return, failure need not be a disgrace. Furthermore, entrepreneurship education should be designed to improve rural dwellers’ self-efficacy beliefs, guide them to have rational outcome expectations, set appropriate goals, and strengthen their career adaptability.

Our research also found that indecisiveness can hinder individual entrepreneurial intention. Since those with limited knowledge and information sources are more likely to be indecisive in their career decision-making process, trainers and educators, as well as policy makers, who aim to strengthen entrepreneurship, should offer supportive information to help better cope with environmental uncertainty and establish self-efficacious beliefs, which can stimulate entrepreneurial intentions (Pérez-López et al., 2019).

Especially for career counselors who expect to develop entrepreneurial careers for their clients, our focus on the mechanisms underlying entrepreneurial intention may provide important angles for an entrepreneurship career. For instance, entrepreneurship training programs should cultivate career adaptability, considering its importance in facilitating entrepreneurial intentions. Courses that focus on self- and environment explorations could be introduced to help entrepreneurs better understand their professional ability and match to potential career opportunities, which are beneficial to developing their career adaptability.

### Limitations and Future Directions

Despite this study’s strengths, we recognize the limitations of its external validity. Obviously, our sample is from a very specific context, and Western countries may have different levels of sensitivity to face consciousness than do East Asians (Begley and Tan, 2001). Bruton et al. (2018) argue that scholars are myopic by relying on Western perspectives of entrepreneurship and should instead examine the variety and diversity of “indigenous entrepreneurship activities” found elsewhere. While we did that, it is equally important that our theoretical perspective be verified in Western cultures. In addition to non-Confucius societies, future studies can also be conducted in urban regions or different political-economic environments. While we proposed and tested the moderating role of indecisiveness on the relationship between face consciousness and career adaptability, there may be other critical variables influencing the career construction processes. For example, social support and barriers may promote or hinder career adaptability (Han and Rojewski, 2014; Pajic et al., 2018). Because the participants self-reported our study variables at one time, we encourage replications to collect multisource and longitudinal data. Having adopted a Chinese version of the existing and widely used scales that are suitable in the Chinese context to measure our variables, we are happy to offer without reservation the data used in our study to readers requesting them for replication.

Finally, previous research has argued that entrepreneurial intention is a critical predictor of entrepreneurial activities (e.g., Krueger et al., 2000; Tsai et al., 2016) but rarely report actual actions taken and measure performance that would better understand the determinants of success. In our study, we used the scale of entrepreneurial intention, which measured some potential entrepreneurial actions of the participants. However, future research should use scales that better reflect actual entrepreneurial behaviors.

## Data Availability Statement

The raw data supporting the conclusions of this article will be made available by the authors, without undue reservation.

## Ethics Statement

The studies involving human participants were reviewed and approved by the Academic Ethics Committee of Southwestern University of Finance and Economics. The participants provided their written informed consent to participate in this study.

## Author Contributions

MT and MM designed the study. MT drafted partially the manuscript and revised it according to reviewer feedback. FL drafted other parts of the manuscript and made data collection. PM framed and revised the manuscript as well as made language editing and helped clarify the table data and figures. MM contributed to writing the theoretical background and testing hypothese. PX verified the data analysis and addressed all the comments related to data analysis. All authors contributed to the manuscript and approved the submitted version.

## Conflict of Interest

The authors declare that the research was conducted in the absence of any commercial or financial relationships that could be construed as a potential conflict of interest.

## Publisher’s Note

All claims expressed in this article are solely those of the authors and do not necessarily represent those of their affiliated organizations, or those of the publisher, the editors and the reviewers. Any product that may be evaluated in this article, or claim that may be made by its manufacturer, is not guaranteed or endorsed by the publisher.
